# Cognitive impairments and predominant polarity in bipolar disorder: a cross-sectional study

**DOI:** 10.1186/s40345-017-0085-5

**Published:** 2017-05-12

**Authors:** Gabriel Okawa Belizario, Alexandre Duarte Gigante, Cristiana Castanho de Almeida Rocca, Beny Lafer

**Affiliations:** Bipolar Disorder Research Program, Department of Psychiatry, University of São Paulo Medical School, Rua Dr. Ovidio Pires de Campos, 785-3º Andar/Ala norte/Ceapesq/Sala 4, São Paulo, SP Zip code: 05403-010 Brazil

**Keywords:** Predominant polarity, Cognitive deficits, Neuroprogression, Bipolar disorder

## Abstract

**Background:**

Bipolar disorder (BD) patients exhibit cognitive impairments during euthymic states. Studies suggest that manic episodes may be correlated to cognitive impairments. The present study investigated the relationship between predominant polarity and the cognitive deficits frequently detected in bipolar patients. We hypothesize that mania predominant polarity (MPP) patients should exhibit greater cognitive impairments in comparison to depressive (DPP) and indefinite predominant polarity (IPP) patients and healthy control (HC) individuals.

**Methods:**

The study evaluated 55 euthymic BD patients, type I and II, and 31 HCs. Patients were divided into 3 groups: MPP (*n* = 17), DPP (*n* = 22), and IPP (*n* = 16), and compared regarding demographic and clinical variables, and performance on a 7-test neuropsychological battery.

**Results:**

MPP patients demonstrated greater cognitive impairments in alternating attention, verbal fluency, and delayed memory in comparison to DPP, IPP, and HC. Compared to HC, IPP patients exhibit cognitive deficits in verbal fluency and alternating attention and DPP patients solely in verbal fluency. Furthermore, DPP patients did not exhibit, in none of the seven neuropsychological tests, significant poorer performances than MPP or IPP patients, although having significant more episodes than MPP patients.

**Conclusion:**

MPP patients exhibit increased cognitive impairments in comparison to DPP, IPP, and HC subjects. Manic episodes may play an important role in the development of cognitive deficits and thus, in potential neuroprogression. Predominant polarity may be an important specifier for predicting future cognitive impairments.

## Background

Bipolar disorder (BD) is a chronic and recurrent psychiatric disease affecting approximately 2.4% of the population worldwide (Merikangas et al. 2011). The disorder affects all ages and genders (Merikangas et al. 2011), and currently represents the highest suicide rate among psychiatric disorders (Goldstein et al. 2012). The bipolar diagnosis is divided into two main categories: the type I, which includes the presence of one or more manic episodes or mixed episodes, and the type II, characterized by recurrent episodes of depression and hypomania.

BD diagnoses can be accompanied by course specifiers intended at providing supplementary information about each patient. Presence of psychotic features, being in partial or full remission, and severity are some of the most commonly used specifiers (American Psychiatric Association 2013). Recent reviews suggest that predominant polarity (PP) may be an important specifier in BD (Carvalho et al. 2014). There is, however, a lack of consensus on a unifying definition of the specifier. The majority of studies utilizing the PP specifier considers a simple definition in which the patient must demonstrate a greater number of episodes, throughout the course of the disease, of a specific polarity (Carvalho et al. 2014). The PP specifier consists of three distinct categories: (1) mania predominant polarity (MPP); (2) depressed predominant polarity (DPP); and (3) indefinite predominant polarity (IPP). Patients with MPP and DPP specifiers exhibit significant differences when compared; DPP has been associated with higher rates of suicide attempts, and MPP, with higher rates of substance abuse and earlier onset of symptoms (Carvalho et al. 2014).

Recent studies encountered pertinent evidence suggesting the presence of neuroprogression in BD patients. The concept of neuroprogression includes measurable impairments in the cognitive and behavioral domains associated with the course of the disorder (Berk 2008). In euthymic bipolar patients, Latalova et al. (2011) found stable and lasting cognitive impairments in the domains of sustained attention, memory, and executive functions. Bourne et al. (2013), in an individual patient meta-analysis, found cognitive impairments, in euthymic bipolar patients, in all 11 measures from the neuropsychological tests California or Rey Verbal Learning Task (VLT), Trail Making Test (TMT), Digit Span, and/or Wisconsin Card Sorting Task, after controlling for IQ, age, and gender. Furthermore, studies suggest that manic episodes may be correlated to the presence of cognitive impairments in bipolar patients. Cavanagh (2002) found a negative correlation between performance in the CVLT test (California Verbal Learning Test) and number of past manic episodes and López-Jaramillo et al. (2010), in bipolar I patients, found a negative correlation between the number of manic episodes and performance on neurocognitive tests. In this study, number of depressive episodes, chronicity of the disorder, age of onset, and medication revealed no relationship to performance in the same tests. Lastly, Murphy et al. (2001) found manic, but not depressed, patients made suboptimal decisions—an impairment that correlated with the severity of their illness.

This study investigated the relationship between the PP specifier and the cognitive deficits often found in bipolar patients. Considering the association of manic episodes to cognitive impairments, and the higher frequency of manic episodes in MPP patients in comparison to depressive episodes, the study hypothesized that MPP patients should exhibit greater cognitive impairments in comparison to DPP and IPP patients and to healthy control (HC) individuals.

## Methods

The clinical group consisted of diagnosed bipolar patients, selected from the outpatient clinic of the Bipolar Disorder Research Program at the Institute of Psychiatry of the University of São Paulo Medical School. Clinical and demographic variables, including the number of episodes and their respective polarities, were collected utilizing the SCID-CV (First et al. 1996). Participants had to present, during the course of the disease, a higher frequency of episodes of one pole in order to be included into either the MPP or the DPP groups. Patients who had the same number of manic and depressive episodes were assigned to the IPP group. Mixed episodes were not considered in this study for determining predominant polarity. The HC group consisted of volunteers devoid of any psychiatric diagnoses. This research has been approved by the Ethical Committee of the Hospital das Clínicas da Faculdade de Medicina da Universidade de São Paulo (Protocol number 793/03), and all participants have signed an informed consent form.

Inclusion criteria to the clinical group required: a diagnosis of BD, type I or type II, in accordance to diagnostic and statistical manual of mental disorders (5th ed.) (American Psychiatric Association 2013); be 18–50 years old of age; attest completion of primary school; and be in an euthymic state for the past 2 weeks, the latter defined by a score lower than 7 in the Hamilton scale for depression—21 items (Hamilton 1960) and in the Young Mania scale (Young et al. 1978). Patients presenting substance-induced disorders, schizoaffective disorder, mental retardation, dependence or abuse of drugs, and/or alcohol in the past 6 months, history of seizures, consumption of benzodiazepines within the past 6 months, or an IQ (intelligence quotient) below 80 were not included in the clinical sample. Inclusion to the HC group required the following: residence in São Paulo; be 18–50 years of age; and a score lower or equal to 5 in the Self Report Questionnaire (SRQ-20) (Harding et al. 1980), which assessed the presence of psycho-emotional disturbances and has been validated in the Brazilian population (Mari and Williams 1986). Individuals presenting any psychiatric disorders, acute and chronic clinical conditions, dependence of alcohol or psychoactive drugs, or an IQ lower than 80 were excluded from the HC group.

All participants were individually submitted to a neuropsychological battery of 7 tests. The tests were selected according to their respective areas of evaluation: attention, verbal fluency, planning, and memory. A single researcher administered all tests and was blind to the clinical status and group of the participants. The neuropsychological tests utilized in this study and their respective domains of assessment were as follows: (1) Stroop Color–Word Test (Spreen and Strauss 1998): assessing mental flexibility and inhibitory control; (2) Verbal Fluency—FAS (Spreen and Strauss 1998): assessing verbal fluency, inhibitory control and the correct use of strategies; (3) Trail Making Test (Spreen and Strauss 1998): assessing alternating attention; (4) Logical Memory I and II (Wechsler 1997): assessing verbal memory; (5) List of Words (Wechsler 1997): Assessing learning and susceptibility to interference in attentional processes; (6) Memory for Scenes (Wechsler 1997): assessing visual memory; and (7) Rey Complex Figure (Rey 1941): assessing planning and problem solving.

The study employed an analysis of variance (ANOVA) to assess the presence of significant differences between demographic variables and the Chi-squared independence test to assess the presence of significant differences between clinical variables. A one-way analysis of covariance (ANCOVA) compared performance between groups in the seven neuropsychological tests and a Fisher’s least significant difference (LSD) post hoc test was conducted, on the variables exhibiting significant effects by the ANOVA, in order to identify which pairs of groups significantly differed among them. The alpha level that defined significance was 0.05 and the analyses were conducted using the IBM SPSS 21.0 statistical package.

## Results

The study evaluated 86 participants in total, 33 males and 53 females (38% and 62% of the total sample respectively). The average age was 37 years old (*SD* = 9.9). The clinical group consisted of 55 patients (64% of the total sample) divided into 3 groups: (1) 17 MPP patients; (2) 22 DPP patients; and (3) 16 IPP patients. The MPP group included 17 bipolar I patients and zero bipolar II patients; the DPP group included 14 bipolar I patients and 4 bipolar II patients; and the IPP group included 9 bipolar I patients and zero bipolar II patients. The HC group consisted of 31 individuals. A one-way between subjects ANOVA (analysis of variance) (Table 1), comparing the effects of predominant polarity on demographic variables (age, years of schooling and estimated IQ) and clinical variables (time since onset and number of episodes), revealed significant results for number of manic [*F*(3,73) = 12.642, *p* < 0.001] and depressive [*F*(3,66) = 20.353, *p* < 0.001] episodes, which was expected due to the method patients were assigned to groups, and total number of episodes, with DPP presenting more past episodes than the other groups [*F*(2,52) = 9.955, *p* < 0.001]. A Chi-squared test of independence (Table 1) was performed in order to examine the association between predominant polarity and psychiatric comorbidities in BD patients. The relationship between these variables was not significant.

**Table 1:** Demographic and clinical variables of MPP, DPP, and IPP, and HC subjects

Characteristics	MPP (*n* = 17)	DPP (*n* = 22)	IPP (*n* = 16)	HC (*n* = 31)	*p* value
Age (years)*	37.94 (10.30)	36.68 (10.11)	39.63 (13.80)	35.35 (6.96)	0.550
Gender (% male)*	47.0	45.4	31.2	32.2	0.606
Age of onset (years)*	23.83 (9.66)	25.00 (5.83)	21.66 (10.78)	–	0.690
Years of schooling*	12.47 (2.57)	12.82 (1.87)	12.63 (1.96)	11.74 (1.98)	0.260
Estimated IQ*	93.35 (8.80)	101.95 (10.25)	99.00 (12.36)	97.23 (9.50)	0.073
Time since onset of illness*	10.50 (8.73)	10.27 (5.96)	11.00 (8.02)	–	0.977
Number of manic episodes*	5.06 (5.14)	2.56 (2.45)	4.00 (2.93)	–	*<0.001*
Number of depressive episodes*	2.07 (1.43)	8.21 (6.62)	4.75 (4.06)	–	*<0.001*
Number of episodes*	7.82 (6.23)	16.59 (10.09)	6.75 (3.99)	–	*<0.001*
Psychotic symptoms**	11 (64.7)	8 (44.4)	3 (37.5)	0 (0)	0.338
Substance dependence**	4 (23.5)	7 (31.8)	2 (12.5)	0 (0)	0.384
Anxiety disorders**	9 (52.9)	11 (50)	6 (37.5)	0 (0)	0.986
Social phobia**	1 (5.8)	3 (13.6)	3 (18.7)	0 (0)	0.534
Specific phobia**	2 (11.7)	0 (0)	0 (0)	0 (0)	0.098
Obsessive–compulsive disorder**	2 (11.7)	3 (13.6)	2 (12.5)	0 (0)	0.984
Post-traumatic stress disorder**	1 (5.8)	0 (0)	0 (0)	0 (0)	0.438

*MPP* mania predominant polarity; *DPP* depressive predominant polarity; *IPP* indefinite predominant polarity; *Control* healthy subjects

The significance for the italic values are for p < 0.05

* Results expressed as mean (SD)—one-way ANOVA

** Results expressed as frequency (%)—Chi-squared test

A one-way between subjects analysis of covariance (ANCOVA) (Table 2), controlling for number of episodes, revealed significant results on the List A [*F*(4,84) = 3.623, *p* = 0.014] and B [*F*(3,84) = 4.662, *p* = 0.002] from the List of Words Test, on the variable Delayed Memory [*F*(3,84) = 3.231, *p* = 0.025] from the Logical Memory I and II Test, on Trail A [*F*(3,84) = 4.829, *p* = 0.011] from the Trail Making Test, and on the Verbal Fluency Test—FAS [*F*(3,84) = 10.646, *p* < 0.001]. The other neuropsychological tests utilized in this study did not exhibit significant results (Table 2). Years of schooling (*p* = 0.105) and estimated IQ (*p* = 0.900), which could influence performance on neuropsychological tests, and gender (*p* = 0.187), due to the greater number of female participants, did not exhibit significance in the Levene’s Test, suggesting homogeneity of the variables among the groups.

**Table 2:** Neuropsychological tests’ scores from bipolar patients with MPP, DPP, and IPP specifiers, and HC

Neuropsychological tests	MPP (*n* = 17)	DPP (*n* = 22)	IPP (*n* = 16)	HC (*n* = 31)	*p* value
Memory for scenes					
Immediate recall	32.38 (11.17)	35.45 (11.10)	30.13 (11.65)	37.65 (10.99)	0.104
Latent recall	30.25 (11.41)	34.50 (12.31)	30.50 (12.13)	37.06 (12.28)	0.138
List of words					
List A	27.00 (5.83)^a^	29.36 (6.79)	27.25 (6.99)^a^	32.26 (5.58)	*0.014*
List B	5.81 (2.45)^a^	7.05 (2.28)	5.50 (2.42)^a^	8.00 (2.50)	*0.002*
Number of trials	4.19 (1.04)	4.59 (1.59)	3.88 (1.50)	4.90 (1.60)	0.135
Logical Memory I and II					
Immediate recall	20.69 (5.91)	22.45 (6.83)	22.75 (5.89)	23.48 (7.47)	0.766
Total recall	32.63 (9.80)	36.91 (10.47)	36.88 (9.08)	38.13 (11.24)	0.503
Delayed Memory	16.94 (5.78)^a,b^	22.41 (7.72)	20.75 (6.28)	23.55 (8.52)	*0.025*
Recognition Memory	23.94 (3.06)	24.45 (3.61)	24.19 (4.62)	24.45 (3.77)	0.766
Stroop Color–Word Test					
Stroop Test, Card 1	23.60 (34.36)	15.86 (3.88)	15.25 (4.02)	12.84 (2.69)	0.120
Stroop Test, Card 2	23.67 (16.45)	19.50 (6.41)	18.31 (5.13)	16.45 (3.84)	0.073
Stroop Test, Card 3	29.80 (7.89)	26.23 (9.211)	33.31 (11.31)	26.84 (8.92)	0.090
Trail Making Test					
Trail A	44.25 (21.03)^a,c^	35.05 (12.24)	33.25 (12.65)	32.13 (10.12)	*0.011*
Trail B	102.60 (40.96)	82.14 (30.17)	95.69 (40.95)	78.71 (32.03)	0.080
Errors trail A	0.07 (0.26)	0.09 (0.29)	0 (0)	0.13 (0.43)	0.689
Errors trail B	0.80 (1.42)	0.50 (1.10)	0.75 (1.48)	0.97 (1.88)	0.971
Verbal Fluency Test—FAS					
Verbal Fluency	28.25 (9.50)^a,b^	35.14 (7.65)^a^	30.88 (5.11)^a^	38.74 (7.52)	*<0.001*
Rey complex figure					
Adequate representation	31.91 (4.39)	33.16 (3.19)	32.00 (3.52)	33.21 (2.92)	0.483
Adequate planning	9.50 (7.14)	12.13 (5.77)	12.50 (10.28)	–	0.588

Results expressed as mean (SD)—one-way ANCOVA

*MPP* mania predominant polarity; *DPP* depressive predominant polarity; *IPP* indefinite predominant polarity; *Control* healthy subjects

Post hoc: ^a^ compared to HC; ^b^ compared to DPP; ^c^ compared to IPP; *p* < 0.05

The significance for the italic values are for p < 0.05

A Fisher’s least significant difference (LSD) post hoc test was conducted, on the variables exhibiting significant effects by the ANCOVA, in order to identify which pairs of groups significantly differed among them. In the Trail A, the post hoc revealed a significant inferior performance for the MPP group in comparison to the IPP [44.25 (21.03); 33.25 (12.69); *p* = 0.015] and HC [44.25 (21.03); 32.13 (10.12); *p* = 0.001] groups. Performance in the Verbal Fluency Test—FAS was significantly inferior for the MPP group in comparison to the DPP [28.25 (9.49); 35.14 (7.65); *p* = 0.013] and HC [28.25 (9.49); 38.74 (7.51); *p* < 0.001] groups, for the IPP group in comparison to the HC group [30.88 (5.11); 38.74 (7.51); *p* < 0.001] and also for the DPP group in comparison to the HC group [35.14 (7.65); 38.74 (7.51); *p* = 0.003]. The List A, from the List of Words Test, revealed significant inferior performances for the MPP group in comparison to the HC group [27.00 (5.83); 32.26 (5.58); *p* = 0.005], and for the IPP group in comparison to the HC group [27.25 (6.99); 32.26 (5.58); *p* = 0.007]; in List B, from the same test, MPP patients performed inferiorly than HC patients [5.81 (2.45); 8.00 (2.50); *p* = 0.002], and IPP patients also performed inferiorly than HC patients [5.50 (2.42); 8.00 (2.50); *p* = 0.001]. Lastly, the variable Delayed Memory, from the Logical Memory I and II Test, revealed an inferior performance from MPP patients in comparison to the DPP [16.94 (5.77); 22.41 (7.72); *p* = 0.039] and HC [16.94 (5.77); 23.55 (8.51); *p* = 0.021] groups.

## Discussion

The results were supportive of the hypothesis made previously to the start of the current study, which predicted greater cognitive impairments in MPP patients in comparison to DPP, IPP, and HC subjects. The results revealed that MPP patients exhibit significant poorer performances, in comparison to the DPP, IPP, and HC groups in alternating attention, verbal fluency, and delayed memory. DPP patients demonstrated poorer performances, in comparison to the HC group only on the Verbal Fluency Test. Furthermore, DPP patients did not exhibit, in none of the seven neuropsychological tests, significant poorer performances than MPP or IPP patients, suggesting that DPP patients who participated in the study are possibly less affected by neuroprogression than MPP and IPP patients. This has occurred even though DPP patients had significantly more episodes throughout the course of the disorder than MPP patients, suggesting that cognitive impairment may be associated with episodes’ polarity rather than number of episodes.

Our study also found that IPP patients exhibited impaired performances in three neuropsychological tests (List of Words Test A and B, and Verbal Fluency Test) when compared to HC. However, in none of the tests, IPP patients exhibited poorer performances than MPP patients, suggesting that IPP patients may have greater cognitive impairments than HC, but less than MPP patients. These results reinforce the hypothesis that manic episodes may play a significant role in the development of cognitive impairments. IPP patients demonstrate a higher proportion of manic episodes on the total number of episodes in comparison to DPP patients and a lower proportion in comparison to MPP patients, which are congruent to the results found in this study.

A possible alternative explanation for cognitive impairments in MPP patients concerns the use of medications. Certain psychotropic medications, such as antipsychotics, are most often prescribed during manic episodes and have been associated to cognitive deficits. The present study did not control for medications, and therefore, the results may have been influenced by it.

The presence of psychotic symptoms, which has been associated with the development of cognitive deficits in schizophrenic patients (Strauss 1993), was not significantly different between the groups in this study, suggesting that manic episodes, accompanied, or not, by psychotic features, may be detrimental to cognitive functioning by itself.

The study is limited due to the relative small sample size of each subgroup, its cross-sectional design, which only enables associations rather than inferring causality between variables, and the lack of assessment on the influence of psychotropic medications on cognitive measures. The concept of predominant polarity utilized in this study is also limited due to the exclusion of mixed episodes as polarity specifiers. Furthermore, due to underpowered comparisons, the negative results may be inconclusive and therefore should be taken with caution.

The study’s overall results suggest that the PP specifier is associated with different patterns of cognitive deficits and that MPP patients are more affected than patients with depressive and indefinite predominant polarities. Although underpowered, these findings are clinically relevant because it provides clinical professionals with a new prognostic tool and enables the development of new therapeutic schedules that addresses the problem of future cognitive deficits. Finally, the PP specifier may be utilized as an instrument to further understand the complex neuroprogression of the disorder.

## Abbreviations

BD: bipolar disorder
PP: predominant polarity
MPP: mania predominant polarity
DPP: depressive predominant polarity
IPP: indefinite predominant polarity
HC: healthy control group
